# Event-Related Potentials for Post-Error and Post-Conflict Slowing

**DOI:** 10.1371/journal.pone.0099909

**Published:** 2014-06-16

**Authors:** Andrew Chang, Chien-Chung Chen, Hsin-Hung Li, Chiang-Shan R. Li

**Affiliations:** 1 Department of Psychology, National Taiwan University, Taipei, Taiwan; 2 Center for Neurobiology and Cognitive Science, National Taiwan University, Taipei, Taiwan; 3 Department of Psychiatry, Yale University, New Haven, Connecticut, United States of America; 4 Department of Neurobiology, Yale University, New Haven, Connecticut, United States of America; 5 Interdepartmental Neuroscience Program, Yale University, New Haven, Connecticut, United States of America; University of Texas at Dallas, United States of America

## Abstract

In a reaction time task, people typically slow down following an error or conflict, each called post-error slowing (PES) and post-conflict slowing (PCS). Despite many studies of the cognitive mechanisms, the neural responses of PES and PCS continue to be debated. In this study, we combined high-density array EEG and a stop-signal task to examine event-related potentials of PES and PCS in sixteen young adult participants. The results showed that the amplitude of N2 is greater during PES but not PCS. In contrast, the peak latency of N2 is longer for PCS but not PES. Furthermore, error-positivity (Pe) but not error-related negativity (ERN) was greater in the stop error trials preceding PES than non-PES trials, suggesting that PES is related to participants' awareness of the error. Together, these findings extend earlier work of cognitive control by specifying the neural correlates of PES and PCS in the stop signal task.

## Introduction

People respond to errors or conflicts by slowing down, a hallmark of cognitive control. The cognitive and neural mechanisms of post-error slowing (PES) and post-conflict slowing (PCS) have been a focus of investigation in numerous studies[1], [2], but continue to be debated to date. For instance, in our earlier work combining functional magnetic resonance imaging (fMRI) and a stop signal task, we distinguished go trials following a go, stop success, and stop error trial. Compared to post-go go trials, both post-stop success and post-stop error go trials are prolonged in reaction time. However, while post-stop error slowing involved activation of right ventrolateral prefrontal cortex (VLPFC), post-stop success slowing did not involve significant activation of a distinct brain region [3], [4]. We hypothesized that, while participants engage an active decision to control response speed during post-stop error slowing, post-stop success slowing may result from a multitude of cognitive processes such that each process alone is not sufficient to drive cerebral responses.

Previous studies of event-related potential (ERP) suggested that the anterior N2 (N200), an ERP that occurs with a latency between stimulus onset and motor response, is related to conflicting goals and prolonged response time (RT) during correct responses, with amplitude greater in high-conflict than low-conflict condition in Stroop and flanker tasks [5], [6]. In the stop-signal task, the amplitude of anterior N2 is larger during go trials following a stop trial, suggesting that N2 reflects frontal control following response conflict [7]. Other studies employing flanker task suggested that the peak latency of N2 increase with RT during conflict trials [8], [9]. Together, these studies point to N2 as an important ERP correlate of conflict and conflict-related cognitive control. However, recent fMRI studies support different neural circuits for error- and conflict-related cognitive control [10], [11], and to our knowledge, no studies distinguished the ERP correlates between post-error and post-conflict slowing within a single behavioral task. The current study aimed to fill this gap of research.

According to the conflict monitoring hypothesis, PES results from cognitive control after detection of an error [12]. Error-related negativity (ERN or Ne), a negative event-related potential (ERP), arises immediately during errors in contrast to correct responses and likely originates from the anterior cingulate cortex (see Wessel, 2012 for a review)[13]. Earlier studies showed that the amplitude of ERN increases with the extent of PES [14]–[17], in support of the conflict monitoring hypothesis [9]. Error-related positivity (Pe), another response-locked ERP component arising slowly following ERN, is related to error awareness and PES. The amplitude of Pe was correlated to PES in a two-choice reaction task [18] and anti-saccade task [19]. In the latter study, Pe was larger when participant were aware of their response error. However, studies have also reported results inconsistent with the conflict monitoring hypothesis. For instance, the amplitude of ERN did not predict the magnitude of PES [18], [19]. An earlier fMRI study of a stop-signal task showed greater activation of the medial frontal cortices (MFC) including the ACC and supplementary motor area during errors and activation of the right VLPFC during PES [3], [4], [11]. However, across subjects, the responses of MFC and VLPFC were not correlated, nor were they correlated with PES, at odds with the conflict monitoring hypothesis.

In this study, we aim to investigate the neural correlates of PES and PCS with the same behavioral paradigm. We employed a stop-signal task, in which participants respond to a go-signal in most trials, and, in parallel, prepare to withhold the motor response when a stop signal appears [20]–[22]. We used a staircase procedure in order to elicit errors in approximately half of the stop trials despite constant behavioral adjustment of the participants. Our goals are two-fold: to identify the neural correlates of PES and PCS; and to examine whether ERN and/or Pe is related to PES.

## Methods

### Participants

Sixteen healthy adults (8 females, 22.4±1.4 years of age, all right-handed, and using their left hand to respond) participated in the study. The participants were all college students of the National Taiwan University, and naïve to the purpose of the experiment. All of them provided written consent and were financially compensated for participation. The use of human participants followed the guideline of Helsinki Declaration and was approved by the Research Ethics Committee of National Taiwan University.

### Behavioral task

**Figure 1:**
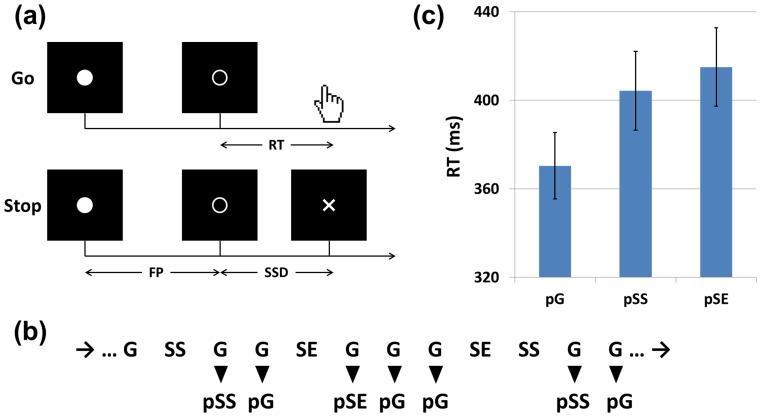
Stop signal task and trial structure. (a) Stop signal paradigm. In “go” trials (∼75%), observers responded to the go signal (a circle), and in “stop” trials (∼25%), they had to withhold the response when they saw the stop signal (an X). In both trials, the go signal appeared after a randomized time interval between 1 to 3 second (the fore-period or FP, uniform distribution) following the appearance of the fixation point. The stop signal followed the go signal by a time delay—the stop signal delay (SSD). The SSD was updated according to a staircase procedure, whereby it increased and decreased by 64 msec following a stop success and stop error trial, respectively. We distinguished go success (G: 98.4±1.7%, mean ± SD) and go error (F: 1.6%), and stop success (SS: 48.6±2.3%) and stop error (SE: 51.4%) trials during the task. (b) An example of trial sequence. Go successes were further distinguished by their preceding trial; thus, G trials preceded by a G, SS, and SE trial were indicated by pG, pSS, and pSE trials, respectively. Depending on whether they increased or did not increase in RT, compared to the mean RT of all preceding pG trials, pSS and pSE trials were further grouped into pSSi and pSSni, and pSEi and pSEni trials, respectively (not shown here; see Methods). (c) Both pSS and pSE trials showed prolonged RT, compared to pG trials, whereas pSS and pSE trials did not differ in RT. Data bars show median RT (mean ± S.E.) across all 16 subjects.

The experimental design and procedures followed that of Li et al. [4] (Figure 1a). We employed a simple reaction time (RT) task of the stop signal paradigm [23]–[25]. There were two trial types, “go” and “stop,” randomly intermixed in presentation and with a ratio of approximately 3 to 1. The inter-trial interval was 2 seconds. A small white dot appeared at the center of a black screen to engage attention at the beginning of every trial. After an interval with a duration ranging randomly from 1 to 3 seconds (the “fore-period”), the dot turned into a circle (∼ 2° of visual angle), which served as a “go” signal. The participants were instructed to quickly press a button at go-signal onset but not before. The circle vanished either at button press or one second after go-signal onset, whichever came first, and the trial terminated. A premature button press before go-signal onset also terminated the trial. In the stop trial, an additional “X”, or the “stop” signal, appeared and replaced the circle after the onset of the go-signal. The duration between the onset of go-signal and the stop-signal, or the stop-signal delay (SSD) was determined by a staircase procedure. The participants were instructed to withhold button press upon seeing the stop-signal. The trial terminated at the button press or one second after the stop-signal onset. The one-up-one-down staircase procedure[26] started at a SSD of 200 ms. The SSD increased and decreased by 64 ms each after a successful and failed stop trial, following our earlier work [4], [27], [28], and many other studies of stop signal task [29]. By increasing and decreasing the stop signal delay each following a stop success and error, the staircase procedure allows participants to succeed in approximately half of the stop trials.

The whole task was divided into 4 sessions, each with 100 trials and lasting no longer than 8 minutes, with a short break in between sessions. There were about 5 minutes of practice prior to the experiments. Participants were trained on the same paradigm before the experiment. The actual number of trials administered varied across individuals, depending on the response times and randomized fore-period. The purpose of training was to ensure that participants understand the behavioral task. The participants were instructed to “respond to the go-signal quickly while watching out for the stop-signal, which might appear in a small number of trials” [4].

### Electroencephalography (EEG) Acquisition

The EEG was collected with a whole-head, 256-channel geodesic EEG system with HydroCell Sensor Nets (Electrical Geodesics, Eugene, OR). This system provides uniform spatial sampling (∼ 2 cm sensor to sensor), covering the entire scalp surface and extending 120° in all directions from the vertex reference electrode. The EEG was amplified at a gain of 1000 and recorded with a vertex physical reference. Signals were digitized at 500 Hz with a 16-bit analog-to-digital converter, which allowed an amplitude resolution of 0.076 µV. The computer administering the task sent a digital trigger to the recording system at the onset of fixation and the go-signal of every trial.

### Data Preprocessing

Artifact rejections were performed off-line as follows. First, the raw data were filtered by a 1–100 Hz band-pass and 60 Hz Notch (FIR) filter. Second, noisy channels, which contained more than 20% of samples exceeding a pre-designated threshold (200 µV), were replaced by the average of the six nearest spatial neighbors. Typically, only two to four channels per session were substituted. Next, for each channel, the EEG epochs that contained a large percentage (15%) of data samples exceeding a threshold (40 µV) were excluded to avoid artifacts related to eye blinks and/or movements. An epoch was defined as the time window between −100 to 1000 ms of the go-signal onset in a trial. Once noisy channels were substituted and artifactual epochs were excluded, the EEG was re-referenced to the common average of all of the channels. Baseline correction was performed by subtracting the mean voltage of a window from 100 ms to 0 ms before a reference time point (go- or stop-signal onset) in each trial for each channel. All the ERP waveforms have been filtered by Butterworth third-order 40 Hz low-pass filter to eliminate high frequency noise for statistical analyses and visualization.

### Topographic Mapping of EEG

We used EMSE Suite software (Source Signal Imaging, San Diego, CA) for topographic mapping. We constructed field maps with spherical spline interpolations based on the method of Perrin et al. [30], which assumes that all points lie on a spherical surface of a constant radius. Laplacian maps were obtained by computing the Laplacian (second spatial derivative) of the spline polynomial before display. The topographies were computed based on the mean current density within a 50 ms time window with contour level in 0.5 µV.

### Data Analysis and Statistics

Following our earlier work (Figure 1b) [4], we first distinguished four main trial types: response (button press) in go trial (G), no response in go trial (F), stop success (SS), and stop error (SE). Involving incongruent goals between the prepotency to respond and the motor intention to withhold the response, a stop trial is thus of higher conflict, as compared to a G trial. G trials were further divided into four categories according to their preceding trial: post-G G trial (pG), post-F G trial, post-SS G trial (pSS), and post-SE G trial (pSE). The post-stop go trials were further divided into those that increased in RT (pSSi and pSEi) and those that did not increase in RT (pSSni and pSEni), in order to distinguish the neural and cognitive processes involved in post conflict/error behavioral adjustment. For instance, we identified the ERP correlates of post-error slowing by contrasting pSEi and pSEni trials and post-conflict slowing by contrasting pSSi and pSSni trials. In another analysis, stop error trials were categorized according to whether their subsequent go trial increased in RT (SEi) or not (SEni). To determine whether a go trial increased or did not increase in RT, we compared its RT to the average RT of all pG trials that preceded it in time during each session. The pG trials that followed each post-stop trial were not considered since the neural/cognitive processes associated with these pG trials occurred subsequent to and could not have a causal effect on the post-stop go trial [4].

We obtained the contrast pSEi – pSEni for each individual participant, to identify ERP's associated with post-error adjustment in RT. Also, we obtained the contrast pSSi – pSSni to identify ERP's associated with post-conflict adjustment in RT. To examine the conflict monitoring hypothesis, we computed ERP components time locked to button press for these contrasts: SEi – G and SEni – G. The contrast ERP waveforms of individual participants were then used for random effect analysis [31] with a two-tailed Wilcoxon signed-rank test. We focused on Fz and Cz because these two channels are most sensitive to ERP components of interest (N2, ERN, Pe), according to previous studies (N2: [8], [9]; ERN: [14]; Pe: [18]). That is, the ERP's are mostly fronto-central in origin (see below). In particular, because Fz and Cz time series were highly correlated (r = 0.90, p<0.001, Pearson regression), we used an alpha of 0.05 to control for false positives. This type I error rate was also supported by false discovery rate (FDR) procedure under dependency[32] with the FDR set at 15%, well within the “reasonable range” of 10 – 20%, as suggested by Genovese et al.[33]. In an exploratory analysis, we also examined Pz. We did not have a specific hypothesis regarding findings on Pz and would treat this as a separate analysis.

## Results

### General Behavioral Performance

Behavioral results are summarized in Table 1. Across subjects, the mean and median go trial RT were 393.6±59.7 (mean ± SD) and 376.8±60.5 ms, respectively, consistent with a right-skewed distribution of RT in an RT task[34] rate of successful stop trials was 48.6±2.3%, suggesting the success of the staircase procedure in eliciting errors in approximately half of the stop trials. The critical SSD was computed by a maximal likelihood procedure on the sequence of all staircase-generated SSDs for each participant, and the stop-signal reaction time (SSRT) was computed by subtracting the critical SSD from the median go RT for each participant[4], based on the race model [29].

**Table 1:** General Behavioral Performance.

go RT (ms)	%go		SSRT (ms)	Critical SSD (ms)
393.6±59.7	98.4±1.7	48.6±2.3	216.0±26.9	160.8±75.4

Statistics of post-SE, post-SS, and post-G trials						
	pSEi	pSEni	pSSi	pSSni	pGi	pGni
Number of trials	21.9±5.1	13.5±5.4	21.1±6.8	13.6±5.0	102.6±9.1	119.7±10.8

Statistics of SE/SS trials				
	SEi	SEni	SSi	SSni
Mean SSD (ms)	192.0±72.1	190.9±79.8	141.6±63.5	122.7±72.3

Note: numbers are mean ± standard deviation.

We examined the behavioral performance of post-stop trials with paired t-tests (Figure 1c). There were significantly more pSS and pSE go trials with RT increase than not (p = 0.01 and p = 0.004, respectively), across subjects. In contrast, there were more post-go go trials that did not show an increase in RT than those that did (p = 0.002). Compared to pG (370.4±61.0 ms), both pSS (404.3±71.3 ms, p = 0.003) and pSE (415.0±70.65 ms, p<0.001) trials were significantly prolonged in RT, but pSS and pSE trials did not differ in RT (p = 0.15). Thus, consistent with earlier studies, there was post-error and post-conflict slowing in the stop signal task.

### Event-Related Potential Analysis

We performed ERP analysis in two epochs: ERP stimulus-locked to the go-signal in pSS and pSE trials and ERP response-locked to the button press in G and SE trials. ERP contrasts are presented in mean ± standard error. ERP results are summarized in Table 2.

**Table 2:** ERP results of post-error slowing (PES) and post-conflict slowing (PCS).

	N2 amplitude	N2 latency
PES (pSEi v.s. pSEni)	pSEi > pSEni	n.s.
PCS (pSSi v.s. pSSni)	n.s.	pSSi > pSSni
ERP results of stop error trials preceding RT slowing (SEi) vs. no-RT slowing (SEni)		
	ERN amplitude	Pe amplitude
SEi vs SEni	SEi < SEni	SEi > SEni

Note: n.s. for non-significant.

**Figure 2:**
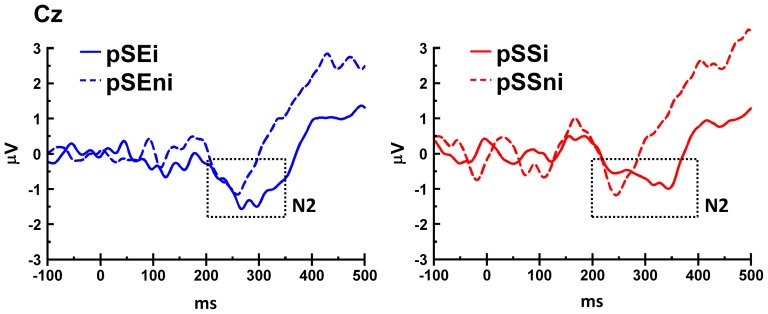
ERP curves during post-stop trials at Cz channel, time-locked to go-signal onset (at 0 ms). Paired signed-rank tests showed that the mean amplitude of N2 was greater in pSEi than pSEni trials (left column), but not greater for pSSi than pSSni trials (right column). On the other hand, signed-rank test showed that the peak latency of N2 was longer in pSSi than pSSni trials, but not longer for pSEi than pSEni trials. These results suggest that increased N2 amplitude was specifically related to post-error slowing, and increased N2 latency was related to post-conflict slowing.

To examine the ERP's during post-stop go trials, we computed the amplitude of N2, time-locked to the go-signal onset (Figure 2), by following these procedures: First, we located the most negative peak in a window of 225 – 350 ms after the go-signal onset, baseline-corrected for a prestimulus period of go-signal. Second, we located the nearest zero-crossing points before and after the peak within a time window 150 – 400 ms after the go-signal onset. Third, we computed the amplitude by integrating the voltage within the two zero-crossing points, which was then divided by the time interval between the two zero-crossing points for each ERP waveform. This allowed us to compute the N2 amplitude while controlling for varying width of waveforms and possible confounding between amplitude and latency. The results showed that N2 amplitude at Cz in pSEi was significantly larger than that in pSEni (p = 0.049, −1.57±0.19 vs. −0.42±0.80 µV), but the same contrast was not significant at Fz (p = 0.96) or Pz (p = 0.13). The N2 amplitude was not significantly different between pSSi vs pSSni trials at Fz (p = 0.41), Cz (p = 1.00) or Pz (p = 0.67). Therefore, the results suggested that N2 amplitude is positively related to PES, but not related to PCS.

We further examined the peak latency difference with the same contrasts. The N2 peak latency difference between pSEi and pSEni was not significant (p = 0.64 at Fz; p = 0.63 at Cz; p = 0.76 at Pz). On the contrary, the N2 peak latency of pSSi is longer than pSSni at Cz (p = 0.04, 199.3±4.6 vs. 184.8±4.4 ms), but not at Fz (p = 0.55) or Pz (p = 0.95). Hence, the results suggested that N2 peak latency is positively related to PCS, but not related to PES.

**Figure 3:**
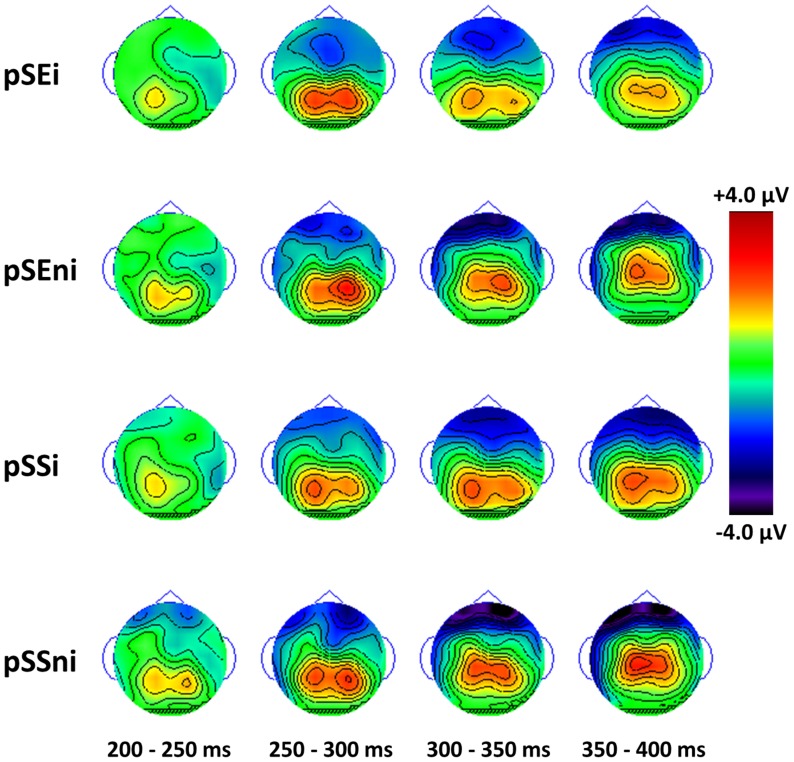
Topographic mapping of N2′s during post-error (pSE) and post-conflict (pSS) trials, stimulus-locked to go-signal onset (at 0 ms) with contour level in 0.5 µV. The N2 of these conditions are frontal-central and approximately symmetric between the two hemispheres.

The topography (Figure 3) shows a negative peak in pSEi, pSEni, pSSi and pSSni trials at the time frame of N2 at the frontal-central region and approximately symmetric between the two hemispheres. Thus, the N2 component identified here is fronto-central in origin, in contrast to the posterior N2 as reported in Folstein & Van Petten [8].

**Figure 4:**
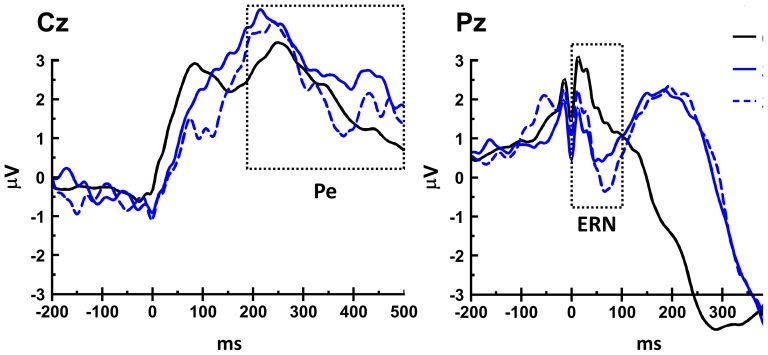
ERP curves during stop and go trials at Cz and Pz channels, time-locked to button press (at 0 ms). The peak-to-peak analysis showed that ERN occurred in both SEi and SEni trials at Pz, compared to G trials. Furthermore, signed-rank test showed that ERN was greater in SEni than SEi trials at Pz. On the other hand, the peak-to-peak analysis showed that Pe occurred in both SEi (at Cz and Pz) and SEni (at Pz), compared to G trials. Furthermore, signed-rank test showed that Pe was greater in SEi than SEni at Cz. These results showed that the amplitude of Pe, not ERN, increased in stop error trials immediately preceding go trials with RT slowing.

The conflict monitoring hypothesis would predict an ERN in the contrast of error and the correct responses. We analyzed the error-related ERP in G and SE trials, time-locked to button press (Figure 4) and baseline-corrected to the mean amplitude of the waveform in the pre-stimulus period of the stop-signal. The ERN amplitude was computed from the ERP waveform averaged across trials for each participant as the difference between the most negative amplitude during the period of 0–100 ms after response and the most positive amplitude during the period -100–0 ms before response, as in Gentsch et al. [35], where positive peak served as baseline correction for the negative peak of ERN. We opted for peak-to-peak measure to quantify ERN under the consideration that it is a sharp waveform with short width and the peak-to-peak method can best capture such a feature. Signed-rank tests between SEi and G trials showed an ERN at Pz (p = 0.02, −2.46±0.29 vs. −1.75±0.39 µV), but not at Fz (p = 0.76) or Cz (p = 0.09). Signed-rank tests between SEni and G trials also showed an ERN at Pz (p<0.001, −3.93±0.54 vs. −1.75±0.39 µV), not at Fz (p = 0.21) or Cz (p = 0.46). Furthermore, a signed-rank test showed a significant difference in ERN amplitude between “SEi – G” v.s. “SEni - G” at Pz (p = 0.006, −0.71±0.26 vs. −2.18±0.40 µV), with the ERN amplitude of SEni greater than SEi. Thus, ERN amplitude does not appear to be positively associated with PES.

We then considered the amplitudes of Pe, which were computed from the ERP waveform averaged across trials for each participant as the difference between the average amplitude in a window from 200 to 500 ms after response and the average amplitude in a window from 100 to 60 ms before [36], [19]. We used this algorithm under the consideration that Pe is slowly rising in magnitude with long width and the averaged amplitude can best capture such a feature. Signed-rank tests between SEi and G trials showed Pe at Cz (p = 0.007, 3.50±0.60 vs. 2.56±0.49 µV) and Pz (p = 0.002, −2.16±0.50 vs. −3.55±0.49 µV), but not at Fz (p = 0.57). But the counterpart signed-rank tests between SEni and G showed Pe at Pz (p = 0.01, −2.42±0.53 vs. −3.55±0.49 µV) but not at Fz (p = 0.23) or Cz (p = 0.12). Furthermore, a signed-rank test showed significant difference in Pe amplitude between “SEi – G” v.s. “SEni - G” at Cz (p = 0.03, 0.94±0.25 vs. 0.34±0.27 µV) but not at Pz (p = 0.47), with the Pe amplitude of SEi greater than SEni. Hence, the results showed that the amplitude of Pe is positively related to PES in the subsequent trial.

## Discussion

### Neural correlates of post-error and post-conflict slowing

In this study, we employed the stop-signal task with the stop-signal delay varied in a staircase procedure to elicit errors in approximately half of the stop trials. In addition to replicating post-error and post-conflict slowing (PES and PCS, [4]), we observed that the amplitude of anterior N2 is positively associated with PES, in a contrast of post-error slowing (pSEi) versus non-slowing (pSEni) trials. Moreover, this difference in N2 amplitude was not observed in the contrast between post-conflict slowing (pSSi) versus non-slowing (pSSni). On the other hand, the peak latency of anterior N2 is positively associated with PCS but not PES. To our knowledge, this is the first report of N2 amplitude and latency as an ERP correlate each specific to PES and PCS. Together, these finding suggest that PES and PCS are associated with different attributes of N2, suggesting potentially different neural mechanisms to mediate post-error and post-conflict slowing.

Anterior N2 has been associated with conflict processing, conflict adaptation and cognitive control, as suggested by many studies [8], . Source estimation showed that anterior N2 is likely to originate from the medial frontal cortex including the anterior cingulate cortex [9], [12], [42], [43]. N2 increases with the degree of conflict between two response units, which delays motor activation and prolongs RT [9], consitent with our finding that N2 is larger in amplitude during PES than non-PES trials. In the stop-signal task, the amplitude N2 is larger during go trials following a stop trial, suggesting that N2 reflects frontal control following response conflict [7]. Also in support is a recent EEG study showing ACC activity during PES [44].

The latency of anterior N2 has been positively correlated to RT in a previous work [8], [9]. Here, we observed this relationship for PCS but not PES. Other studies of the stop-signal task showed that the latency of antrior N2 is related to the stimulus evaluation speed of stop-signal[8], suggesting that post-conflict slowing might result from slower buttom-up processing of stimulus. This is in contrast to PES, which engage a top-down mechanism such as a decision to slow down. broadly consistent with the idea that perceptual and response conflict each induces PCS and PES [2], [45]. However, more recent work of model-based fMRI suggests that both PES and PCS result from a trial-by-trial update of the probability of stop signal [46]. Future studies with a larger sample size may help address these alternative hypotheses.

The current results should be considered along with two previous ERP studies, which did not find amplified N2 related to PES. Beyer et al.[47] employed a modified stop-signal task and showed PES with stimulus or response repetition but not in no-repetition condition; however, N2 amplitude did not differ with respect to previous trial type (stop success, stop error and go). In another study employing stop-signal task with the go stimulus repeated or not repeated, PES occurred in the repeated condition but not in association with increased N2 amplitude [7]. Neither of these studies, however, differenciated post-error trials with or without RT slowing, which may be critical in identifying changes in N2 amplitude.

In summary, PES is positively related to N2 amplitude while PCS is positively related to N2 latency. This differenece in neural responses suggests different cognitive mechanisms for PCS and PES, in accord with previous studies that posited different domain-specific mechanisms invloved in perceptual and response conflict adjustment [2], [45] and fMRI studies showing distinct brain activations to error- and non-error-related cognitive control[10].

### Conflict monitoring hypothesis

Pe amplitude is greater during the stop-error trials preceding slowing (SEi) than non-slowing go trials (SEni), suggesting that this ERP is related to PES. In contrast, ERN does not appear to be positively associated with PES in the current results, and this lack of a correlation between ERN and PES was observed in many previous studies [19], [48]–[51]. For instance, in an anti-saccade task, the amplitude of Pe but not ERN is associated with PES [19]. This dissociation between ERN and PES has also been shown in studies with pharmacological manipulations that affected ERN but not PES [52]–[56] and in clinical conditions that influenced ERN but not PES or Pe [57], [58].

ERN, which occurs immediately after the error response, and Pe, which comes later than ERN with a slower rise in magnitude, appear to have different functional significance in cognitive control. Pe reflects error awareness and strategic adjustment, while ERN reflects implicit processing of errors [19], [48], [59]–[61]. For instance, Nieuwenhuis et al. [19] observed in an anti-saccade task that the mean amplitude of Pe but not ERN is larger in the trials in which participants were aware of errors than when they were not. Importantly, RT tends to be more prolonged following error-aware trials than error-unaware trials. Another ERP study in which subjects rated response correctness reported both Pe and ERN when participants make and perceive errors [59]. However, Pe also occurs when participants are unsure about the correctness even when they actually make a correct response. Together, these results suggest that ERN reflects the actual mismatch between expected correct response and error, while Pe reflects the subjectively perceived mismatch [59]. In a recent ERP study employing a discrimination task with participants adopting a high or low criterion to signal their errors, the amplitude of Pe (but not ERN) increased with a higher criterion but did not vary with the accuracy of reported performance error (categorical decision of performance). This latter finding suggests that Pe reflects participants' detection of error (input) instead of reaching decision (output) [60]. More recently, in speed-accuracy trade-off during perceptual discrimination, the amplitude of Pe is smaller in conditions where the evidence for an error is weaker, consistent with the idea that Pe reflects accumulated evidence for error awareness [61]. Along with these earlier studies, the current finding suggest that PES is related to awareness and not just implicit detection of error.

### Limitations of the study

Our sample size is very small. Thus, although the analyses focused on Fz, Cz and Pz leads on the basis of the literature, the current study may not be powered to observe differences in other locales not examined in the current work. Furthermore, one is to note that the current findings are only marginally significant and that more studies are needed to replicate the current findings and to fully address the issue of multiple comparisons. Secondly, we did not reconstruct the source of N2 or Pe. Thus, the discussion of these ERP correlates with reference to the medial frontal cortex is speculative.

### Conclusions

In conclusion, the current study identified anterior N2 as an ERP correlate of post-error slowing (PES) and post-conflict slowing (PCS). N2 amplitude may reflect participants' strategic adjustment in go trial response following an error. Furthermore, error-related Pe occurs only in error trials that precede PES. In contrast, PCS is related to prolonged N2 peak latency, suggesting that different cognitive mechanism are involved RT slowing in the stop signal task. These results extend earlier ERP studies of cognitive control by substantiating a set of events leading to error/conflict-related behavioral adjustment in the stop signal task.
