# Correction: Event-Related Potentials for Post-Error and Post-Conflict Slowing

**DOI:** 10.1371/journal.pone.0111147

**Published:** 2014-10-09

**Authors:** The PLOS ONE Staff

The second and third sentences of the first paragraph of the General Behavioral Performance section of the results were incorrectly merged. The correct sentences are: Across subjects, the mean and median go trial RT were 393.6±59.7 (mean ± SD) and 376.8±60.5 ms, respectively, consistent with a right-skewed distribution of RT in an RT task [34]. The rate of successful stop trials was 48.6±2.3%, suggesting the success of the staircase procedure in eliciting errors in approximately half of the stop trials.

Table 1 and Table 2 are incorrect. The correct Table 1 and Table 2 are below.

**Table 1:** Summary of Behavior Performance

General Behavioral Performance				
go RT (ms)	%go	%stop	SSRT (ms)	Critical SSD (ms)
393.6 ± 59.7	98.4 ± 1.7	48.6 ± 2.3	216.0 ± 26.9	160.8 ± 75.4

Statistics of post-SE, post-SS, and post-G trials						
	pSEi	pSEni	pSSi	pSSni	pGi	pGni
Number of trials	21.9 ± 5.1	13.5 ± 5.4	21.1 ± 6.8	13.6 ± 5.0	102.6 ± 9.1	119.7 ± 10.8

Statistics of SE/SS trials				
	SEi	SEni	SSi	SSni
Mean SSD (ms)	192.0 ± 72.1	190.9 ± 79.8	141.6 ± 63.5	122.7 ± 72.3

Note: numbers are mean ± standard deviation

**Table 2:** Summary of ERP Results.

ERP results of post-error slowing (PES) and post-conflict slowing (PCS)		
	N2 amplitude	N2 latency
PES (pSEi v.s. pSEni)	pSEi> pSEni	n.s.
PCS (pSSi v.s. pSSni)	n.s.	pSSi> pSSni

ERP results of stop error trials preceding RT slowing (SEi) vs. no-RT slowing (SEni)		
	ERN amplitude	Pe amplitude
SEi vs SEni	SEi <SEni	SEi> SEni

Note: n.s. for non-significant.

Figure 4 is incorrect. The authors have provided a corrected version here.

**Figure 4:**
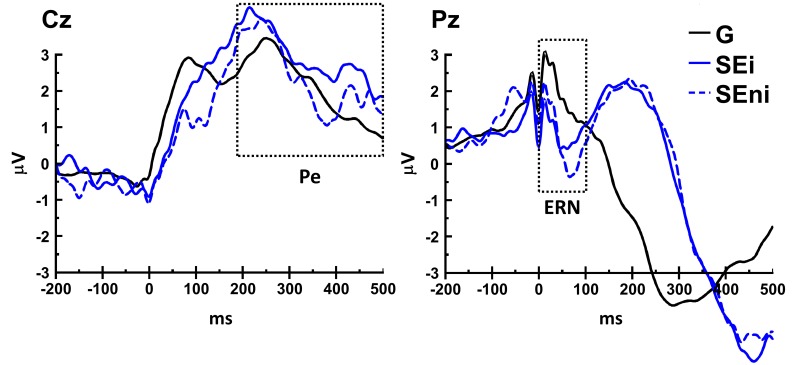
ERP curves during stop and go trials at Cz and Pz channels, time-locked to button press (at 0 ms). The peak-to-peak analysis showed that ERN occurred in both SEi and SEni trials at Pz, compared to G trials. Furthermore, signed-rank test showed that ERN was greater in SEni than SEi trials at Pz. On the other hand, the peak-to-peak analysis showed that Pe occurred in both SEi (at Cz and Pz) and SEni (at Pz), compared to G trials. Furthermore, signed-rank test showed that Pe was greater in SEi than SEni at Cz. These results showed that the amplitude of Pe, not ERN, increased in stop error trials immediately preceding go trials with RT slowing.
